# A comprehensive laser image dataset for real-time measurement of wheelset geometric parameters

**DOI:** 10.1038/s41597-024-03288-y

**Published:** 2024-05-06

**Authors:** Yuezeng Song, Zhenyan Ji, Xiaoxuan Guo, Yihan Hsu, Qibo Feng, Shen Yin

**Affiliations:** 1 School of Software Engineering, BeijingJiaotong University, Beijing, 100044 China; 2 School of Physical Science and Engineering, BeijingJiaotong University, Beijing, 100044 China; 3 Department of Mechanical and Industrial Engineering, Norwegian University of Science and Technology, Trondheim, 7034 Norway

**Keywords:** Mechanical engineering, Electrical and electronic engineering

## Abstract

Railway transportation has experienced significant growth worldwide, offering numerous benefits to society. Most railway accidents are caused by wheelset faults so it’s significant to monitor wheelset conditions. Therefore, we need to collect wheelset images, repaint them, extract laser stripe centerlines, construct 3D contour, and measure their geometric parameters to judge the wheelset’s conditions. Deep learning can fulfill the tasks satisfyingly because it’s adaptable, robust, and generalize compared with traditional methods. To train the deep learning models effectively, we need rich and high-quality wheelset datasets. So far, there are no applicable public train wheelset datasets available, which greatly hinders the research on train wheelsets. Thus we construct a publicly available Wheelset Laser Image Dataset (WLI-Set). WLI-Set consists of four sub-datasets, Original, Inpainting, Segmentation, and Centerline. The dataset contains abundant annotated multiline laser stripe images that can facilitate the research on train wheelsets effectively.

## Background & Summary

Railway transportation has experienced rapid growth in recent decades, significantly benefiting society^1^. According to statistics from the US Department of Transportation, the railway freight volume in the United States reached 26 trillion ton-miles in 2019. During the period from 2006 to 2014, there was a remarkable 50% growth in Australia railway freight^2^. Mainland China has constructed no fewer than 37 900 km of high-speed railway lines crisscross the country since 2008. The network is under quick expansion, and is expected to double again by 2035^3^.

With the development of railway construction, the freight capacity of railways has been significantly enhanced. Freight transport is progressively shifting towards heavy-haul train. Investigations indicate that wheelsets of heavy-haul train exhibit faster and more severe worn than regular train. As the primary interface between the train and the track, wheelsets play a crucial role in maintaining stability. Wheelset-related failures, such as oversized wheel dimensions, not only cause damage to railway infrastructure and the vehicle’s structure itself but also pose significant hazards to railway transportation safety. The Eschede^4^ train derailment accident resulted in 101 fatalities and 88 injuries, making it the deadliest railway accident in Germany. The accident was caused by the rupture of one of the wheels due to metal fatigue, which ultimately led to the derailment of the train. In 2009, the United Kingdom (UK) rail infrastructure manager, Network Rail, spent £32 million on recovering from faults^5^.

**Fig. 1:**
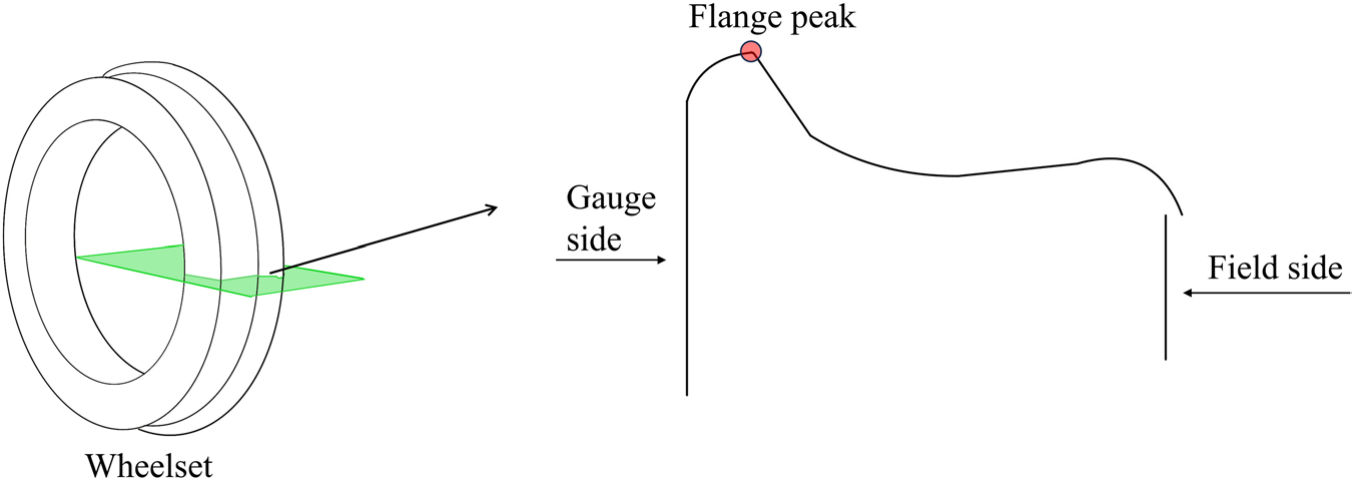
Visualization of the wheelset images and their cross-sectional view. The geometric parameters such as flange thickness, flange height, and wheel diameter can be calculated from the contour line.

By measuring the wheelsets’ geometric parameters, such as the height and thickness of flange shown in Fig. 1, we can timely detect the condition of wheel wear, when these parameter values exceed the safety range, it can be concluded that the wheelset has worn out. And the parameters indicate wheelsets’ lifecycle. Hence, the real-time measurement of wheelset geometric parameters has become a hot topic research among experts and scholars. Conventional measurement methods^6,7^ rely on manual measurements and regular maintenance for assessing wheelsets’ geometric parameters. However, this approach is labor-intensive, resource-consuming, and lacks precision, making it inadequate to meet the requirements of modern railway transportation. Currently, the most popular approach is image-based measurement methods. To be specific, these methods utilize industrial cameras to capture images of the wheelsets, and employ image processing algorithms to refine the images, then we can decouple the geometric parameters of the wheelsets from these refined images. Recent studies have indicated that the most commonly employed method for capturing images of wheelsets is by using lasers and Charge-coupled device(CCD) cameras. Dubnishchev *et al*.^8^ utilized the chord principle to accomplish the measurement of wheelset axle support and wheel diameter with non-contact laser emitters and a camera. Zhang *et al*.^9^ achieved high-precision measurement of wheelsets with two-dimensional laser displacement sensors (2D-LDS). Zheng *et al*.^10^ employed three 1D-LDS instead of a 2D-LDS and applied the three points principle for wheelset measurement.

However, we cannot directly decouple the geometric parameters from the captured images, this is because wheelset laser images are captured in outdoor conditions, diffuse reflection of light and environmental interference result in significant region adhesion (spots) and partial fractures in the laser stripe images. These imaging defects significantly impact the measurement of geometric parameters and surface analysis of the wheelset. It is necessary to remove any spots and fractures to proceed the measurement. With the development of computer vision, researchers started utilizing deep learning to accomplish this task. Zhang *et al*.^11^ proposed a real-time system for measuring the geometric parameters of wheelsets based on laser sensor, CCD and image processing. Ji *et al*.^12^ proposed the Recurrent Similarity Mapping Network (RSM-Net) to restore captured laser stripe images and achieved excellent results on industrial images. Kim^13^ utilized multiple successive image processing algorithms to measure the lateral displacement of wheelsets in a non-contact manner.

The trained deep learning model can perform real-time repair of the wheelset laser images, generating inpainted images that can be used to calculate geometric parameter for analysis. Training deep learning models requires abundant datasets, which providing vital foundation for image processing algorithms. However, to the best of our knowledge, there is currently no publicly available dataset of wheelset laser images for trains. Possible reasons for this phenomenon include: (1) Laser wheelset image data may contain sensitive information about railway systems; (2) Building and maintaining such a dataset requires significant human and financial resources; (3) Specialized equipment and technical expertise are needed. Therefore, we have constructed the Wheelset Laser Image Dataset (WLI-Set), which consists of four subsets, and each subset comprises 2710 laser stripe images of the wheelset. The dataset includes original captured images, inpainted images, segmented ground truth images and centerline images, providing support for the complete pipeline of processing algorithms in wheelset laser images.

Specifically, we adopt the three points principle^10^ to document wheelset laser images. Differing from Zheng, we utilize only two laser emitters and substitute a positioning sensor in place of the emitter right below the wheel. The area array CCD camera and its corresponding multi-line laser emitter are installed on both sides of the track. When the wheel passes the positioning sensor, the CCD cameras take pictures containing multiple laser-line at the same time. The captured images have a resolution of 1280 × 1024 pixels, with large areas of blank space. To improve the image processing results, researchers cropped the images into 800 × 672 pixels, which serve as the original image section of this dataset. After that, researchers annotated the original images by Photoshop. More specifically, our annotation team employed pixel-level brushes and performed the following steps: (1) Erased the light spots and subtracted them from the original image to obtain segmented spot defects, labeling their RGB values as 1. (2) Repaired the broken light stripes and subtracted them from the original image to obtain segmented fracture defects, labeling their RGB values as 2. (3) Added the two defect images together to form the defect segmentation ground truth (GT) image. Following that, researchers conducted comparative captures under low-light conditions, ensuring strict consistency in train status, equipment, and installation position. This yielded a set of repaired images, serving as GT for the complete wheelset laser images. Additionally, researchers used the industrial software HALCON to extract the centerline image of the laser wheelset.

## Methods

**Fig. 2:**
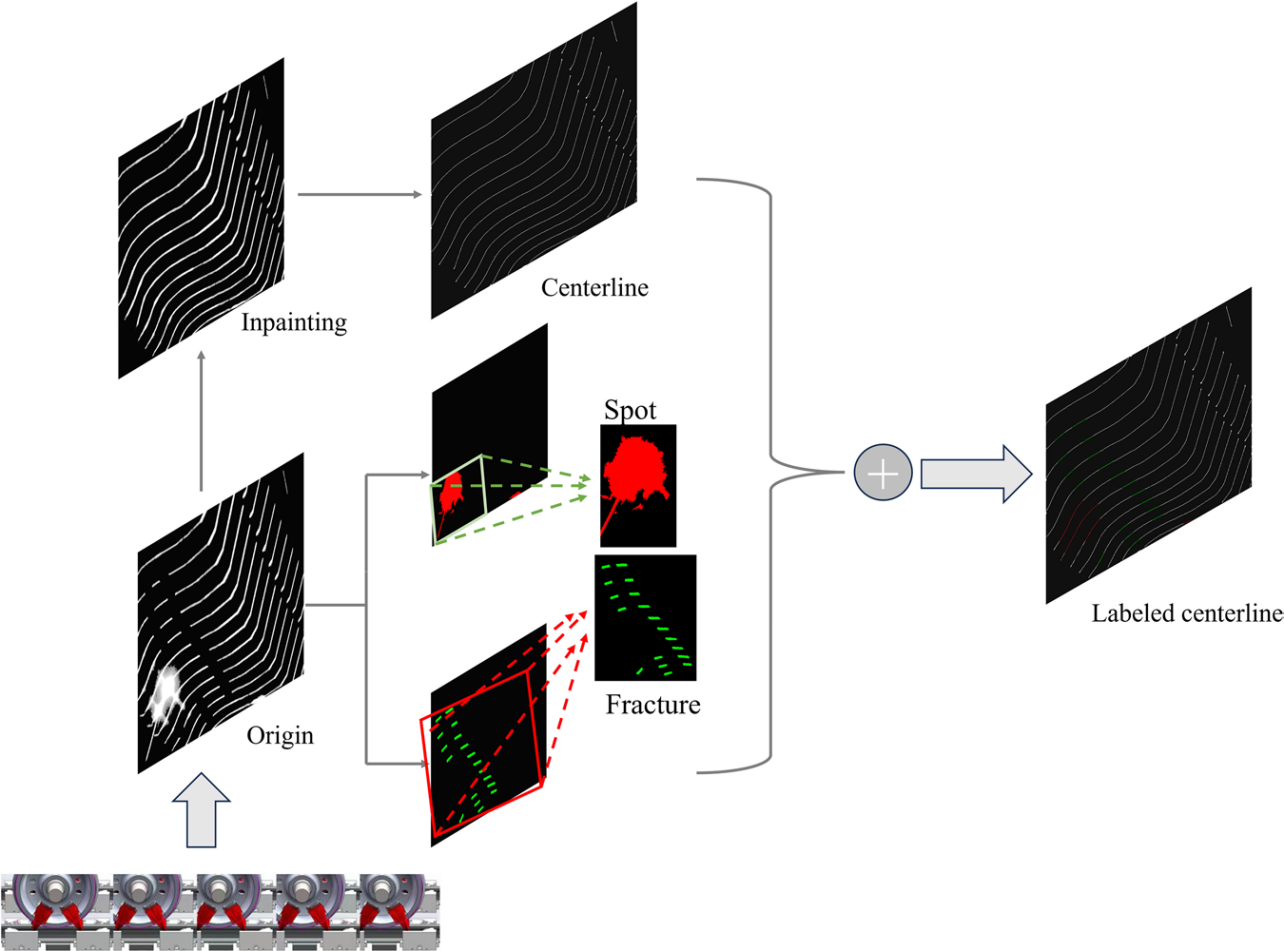
The lower left corner is a schematic diagram of the process of taking a wheel pair laser light strip with a CCD camera. The original image obtained is manually annotated and repaired, and the centerline is extracted. The annotated centerline image is obtained by adding the images.

The dataset proposed in this paper consists of four parts: *origin*, *label*, *inpainted*, and *centerline*. The original images, label images, and inpainted images can be used to train and validate the deep learning model for repairing wheelset images, the centerline images can be used to test and compare the effectiveness of centerline extraction algorithms. These models and algorithms are the core components of the wheelset parameter measurement system. The process of constructing this dataset is illustrated in Fig. 2. We obtained the original images using industrial cameras, then annotated the “fracture” and “spot” regions in the original images, and manually eliminated them to obtain the “inpainted” images. Finally, the centerline images were extracted from the inpainted images. According to the pipeline used to create this dataset, this paper will sequentially introduce the production process from the following three parts.

### Original image

**Fig. 3:**
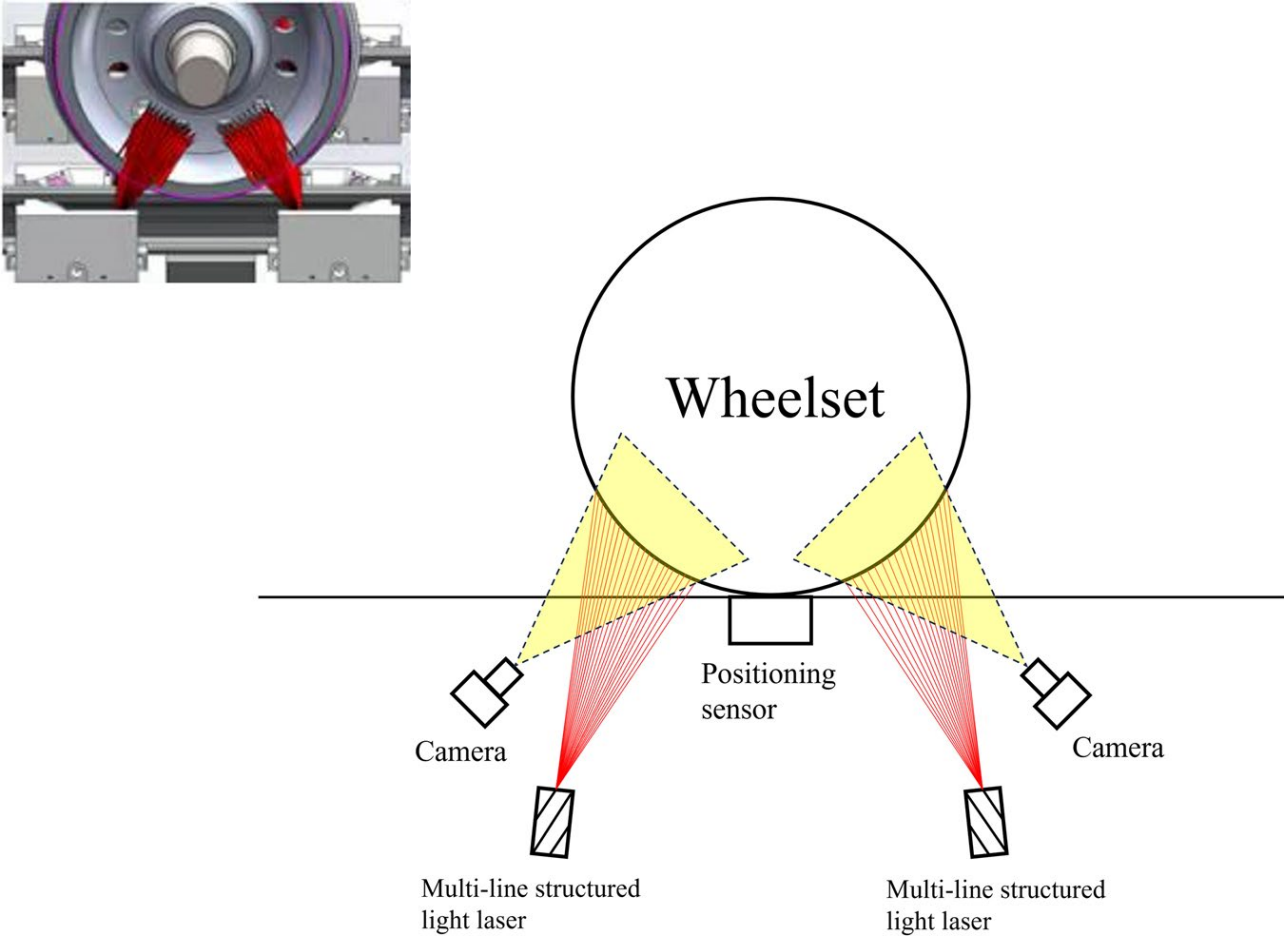
Multi-line laser sensors are placed on both sides of the track. When the train passes by at high speed, the corresponding CCD camera continuously takes multiple laser stripe pictures.

We employed a real-time measurement approach by installing the camera systems on both the inside and outside of the track. When a wheel passes over the system, the sensors simultaneously trigger the inner and outer cameras to capture images, each camera takes a single image. This set of actions repeats when the next wheel passes, and continues until all wheels have passed through the system. The camera system is primarily composed of three components: laser emitter, sensor, and area scan industrial camera. The industrial camera we use is MV-CU120-10GM/GC(NPOE). This camera uses a Sony IMX304 CMOS chip, which has low noise, high resolution, and excellent images, with a maximum frame rate of 9.4 fps at full resolution. This makes real-time transmission of uncompressed images possible. Before each capturing session, we calibrated the camera relied on^14^ for extrinsic and intrinsic calibration. In Fig. 3, the area array CCD camera of the wheelset monitoring equipment and its corresponding multi-line laser sensor array are installed on the left and right sides of the wheelset. The laser emitter emits multiple structured-light rays. When a wheel passes through, the light rays project stripes onto the surface of the wheelset, and at this moment, the CCD camera captures an image, recording a grayscale image of the multi-line laser stripes. When the entire train passes through the sensor equipment, these cameras quickly takes hundreds of photos of the wheelset shape reflected by the multi-line laser and transmits them to the terminal device for real-time storage. However, a single camera can only capture one image when a wheel passing through, resulting in a low sampling frequency. To address the issue, we collected images of heavy-haul freight trains from multiple routes in China, including Fuzhou, Chengdu, Suzhou, and Shenyang, to ensure a sufficient scale of dataset.

Still, the captured image size is 1024 × 1280, which is too large and contains redundant black blank parts. As the available feature information is concentrated in a certain region of the image, we cropped the photo to a size of 800 × 672, only containing the geometric and semantic features of stripes.

### Defect label

**Fig. 4:**
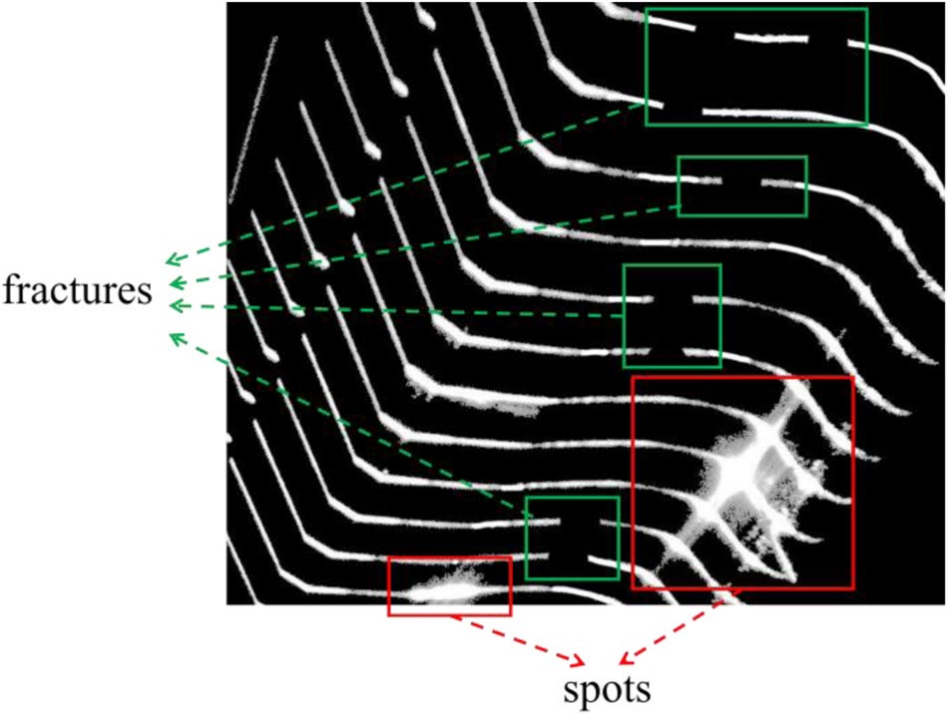
The red box in the picture represents the spot, and the green box in the picture represents the fracture.

Since the equipment is installed in an outdoor environment, the imaging process is affected by environmental interference and reflected light, resulting in images containing small laser-stripe smudging(spot) and missing(fracture). These imaging defects significantly impact the measurement of geometric parameters and surface analysis of the wheelset, it is necessary to remove these defects and calculate the geometric parameters from them. To assist researchers in training deep learning models capable of removing imaging defects, we provide label images in our dataset. As marked in Fig. 4, we divide these defects into “spots” and “fractures”. Our annotation methodology was carefully designed to display defects of stripes. All pictures are annotated manually.

In order to ensure pixel-level annotation, we adopt a special annotation method that differs from most segmentation datasets: extract first and then annotate it. We use Photoshop CS6 to erase spots and connect the fractures of laser-stripes. Photoshop can provide brushes as small as one pixel in size. For large spots, we use a 10–15 pixel brush to apply the background color, and for the fine edges of the spots, we use a 1 pixel brush. The width of the light strip is between 5–13 pixels, and the width of the crack part is basically 8–9 pixels, so we use an 8-pixel brush to supplement the fractures. In order to ensure that the spots are completely applied and the fractures are meticulously connected, we require researchers to annotate for no less than 3 minutes. The annotated images will undergo unified quality control to ensure the pixel-level accuracy of the labels.

Subsequently, the method of image subtraction g(x, y) = |f_1_ (x, y)−f_2_ (x, y)| was employed to subtract the processed image obtained from Photoshop (referred to as PS) from the original image. In the given formula, (x,y) represents the position of a pixel within an image, f_1_, f_2_ and g denote the pixel sets of the original image, modified image, and defective image, respectively. This step yielded two separate images: one containing only the spots, with pixel values limited to (0, 0, 0) and (255, 0, 0), and the other containing only the fractures, with pixel values limited to (0, 0, 0) and (0, 255, 0). Then we use code to annotate the pixels with the value (255, 0, 0) as 1, the pixels with the value (0, 255, 0) as 2, and the remaining pixels as 0.

**Fig. 5:**
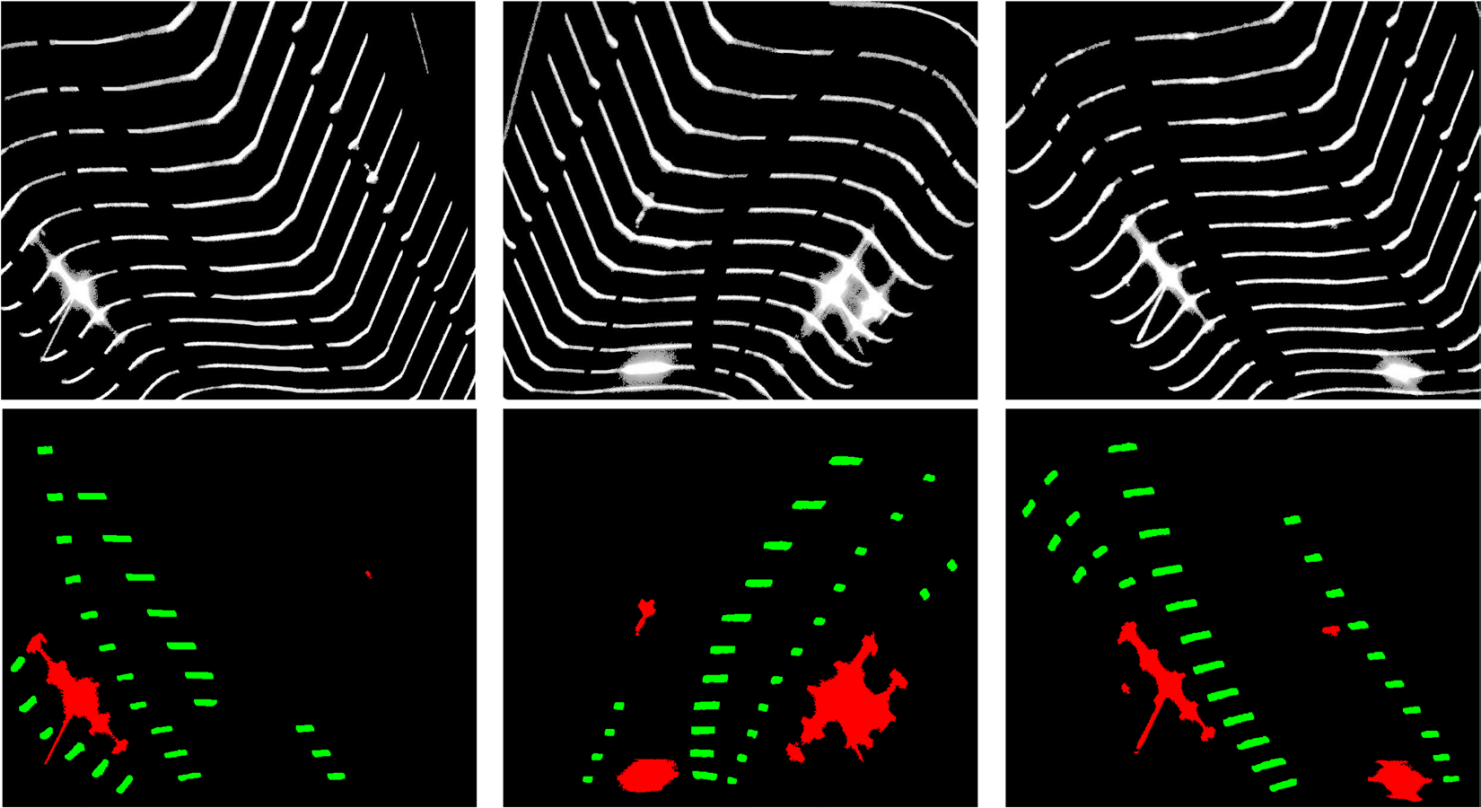
This figure illustrates the annotated examples of defects in the dataset, where the green area represents fractures, and the red area represents spots of light.

Finally, by adding the two classes of labeled images together, the complete label dataset is obtained, as demonstrated in Fig. 5. The formula we utilize is as follows: p(x, y)=q_1_ (x, y) + q_2_ (x, y), where p represents the label image, q_1_ and q_2_ represent the spot defect image and the fracture defect image respectively.

### Inpainted image and centerline extraction

**Fig. 6:**
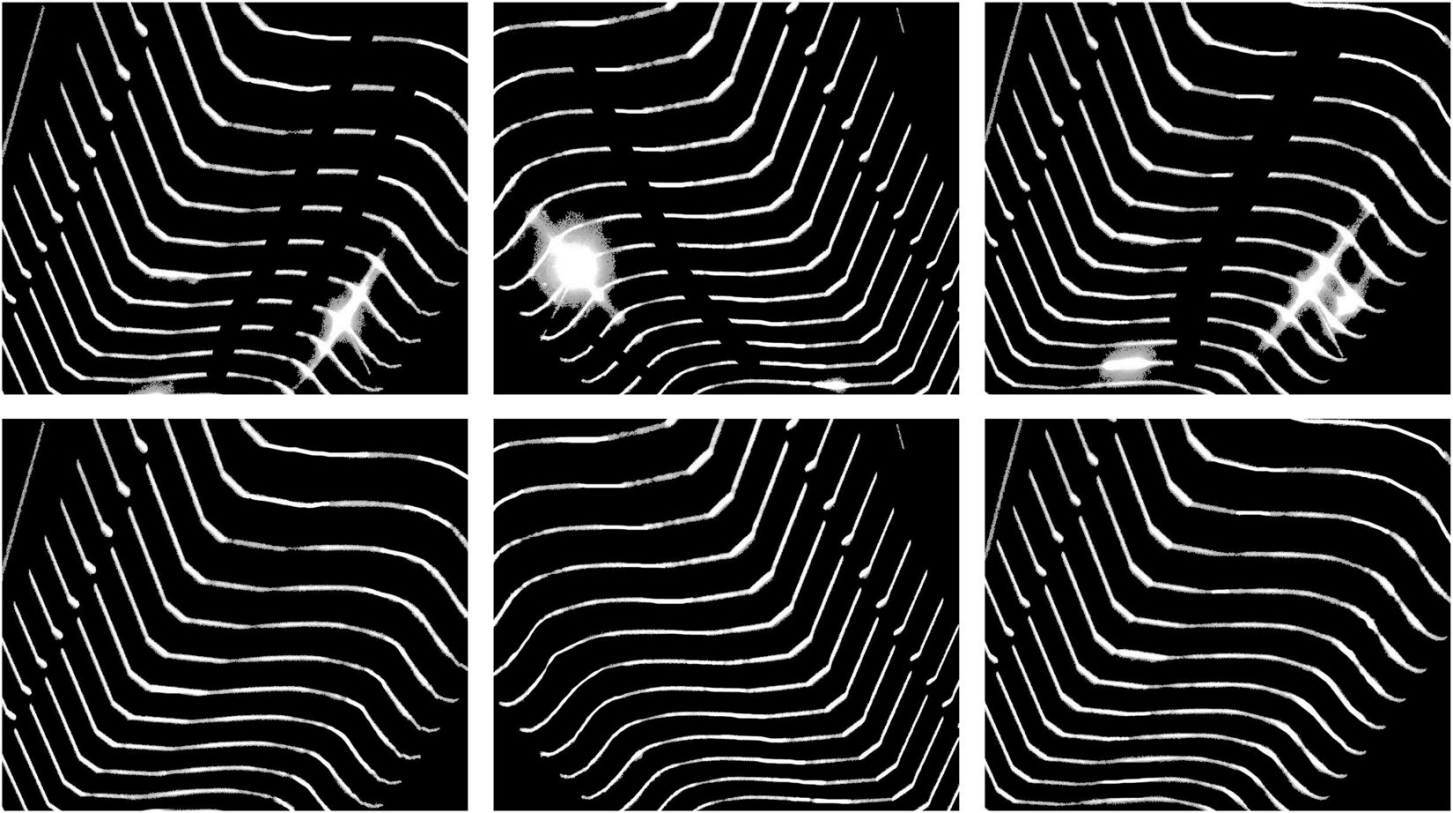
The top row of images represents the captured original images, while the bottom row shows the images after manual inpainting.

It is difficult to collect ideal defect-free images in an industrial environment,therefore, we manually removed the spots and supplemented the missing parts of the fractures using Photoshop. In Fig. 6, Both the spot and fracture parts have been removed, and the resulting image, after centerline extraction, can now be used to measure the geometric parameters of the wheelset. The inpainted images provided in our dataset can be used to evaluate the performance of the deep learning model, and the centerline images can be used to test and compare the effectiveness of centerline extraction algorithms.

**Fig. 7:**
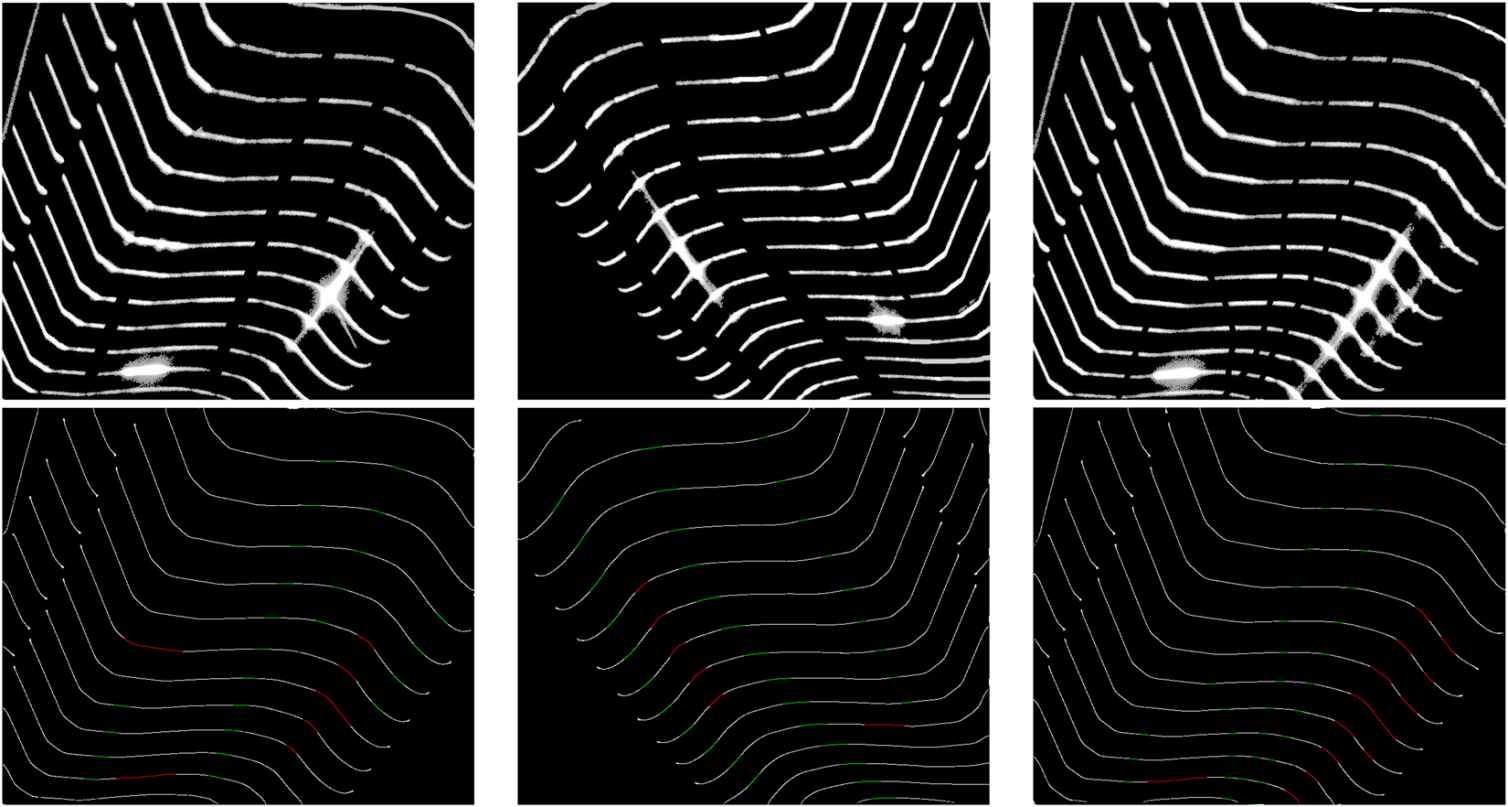
The top row of images represents the captured original images, and the images in the bottom row represent the laser stripe images obtained by extracting the centerline and adding them to the label images. The red portions indicate spots, while the green portions represent fractures.

Due to the limitations of the hardware equipment, the structure-light projected by the laser typically has a width of 3 to 12 pixels. This is not applicable for precise measurement of wheelsets’ geometric parameters. To use it in subsequent measurement, it is necessary to extract the center of the light stripe at the single-pixel or even sub-pixel level. The ideal brightness distribution of the laser stripe’s cross-section follows a Gaussian model. Therefore, the brightest pixels on the cross-section can be extracted, forming the centerline of the laser stripe. We use the HALCON (21.11) software developed by German MVTec Company for the centerline extraction step. The core algorithm for centerline extraction in HALCON is the line_gauss operator. Its main function is to extract lines from images, and the extracted results belong to sub-pixel accurate eXtended Line Descriptions (XLD) contours. At this point, the pixel RGB values in the image are (0, 0, 0) and (255, 255, 255). Similarly, we annotated (0, 0, 0) as 0 and (255, 255, 255) as 1, resulting in a dataset of 2710 centerline images. Then, we added the label images to the extracted centerline images, resulting in the centerline images shown in the bottom row of Fig. 7. As shown in Fig. 7, we have the inpainted image and the centerline image. Experts can calculate the geometric parameters of the wheelset from this fine and precise contour line.

## Data Records

**Fig. 8:**
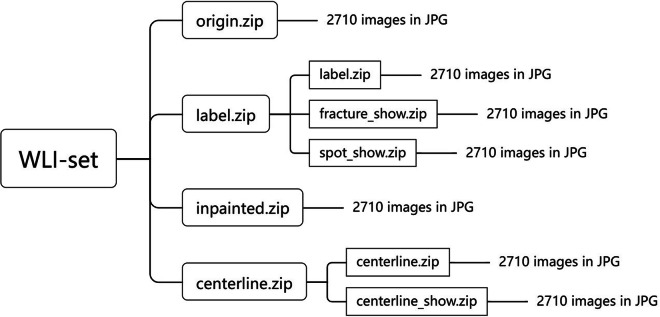
Repository structure of our dataset.

The dataset^15^ were made available from 10.6084/m9.figshare.25040747.v3. This dataset comprises four zip files: Origin.zip, Label.zip, Inpainted.zip and Centerline.zip. The \emph{Origin} comprises 2710 origin monochrome laser-stripe images. The *Label* comprises a collection of 2710 strip defect GT images that we meticulously annotated. To facilitate the use in training deep learning models, the RGB values in ‘label’ images are 0, 1, and 2, making it difficult to distinguish them visually. So, we provide label images with richer colors to enhance visual appeal, the spot labels are painted red in images and the fracture labels are painted green. the Inpainted represents the inpainted GT images we obtained under relatively ideal conditions, and the Centerline are extracted centerline images by a industrial software, we also provide colorful centerline images to assist researchers in visualizing. As shown in Fig. 8, the repository structure of our dataset is presented. All these images are in JPG format.

## Technical Validation

### Reliability of annotation

This paper validates the reliability of image annotations from three aspects: annotation accuracy, dataset distribution balance check, model validation, and comparison.

#### Annotation accuracy

To ensure the accuracy of segmentation masks in the dataset, the reviewers manually inspected all Label image data to verify if the annotations corresponded appropriately to the image content. If any annotation errors were identified, they were corrected accordingly. The examining results indicate that there were no errors in the annotations, and they perfectly matched the image content.

#### Balance check

The purpose of the dataset distribution balance check is to ensure a relative balance in the sample quantities across different categories within the dataset, thus avoiding the model’s excessive preference for certain classes. After inspection, each category consists of 2710 samples. Within the “spot” category, the pixel value differences between samples do not exceed 500, accounting for approximately 3.4% of the total. Within the “fracture” category, the pixel value differences between samples do not exceed 200, accounting for approximately 2.5% of the total. This suggests that the data annotations exhibit distribution balance.

#### Model validation and comparison

There are differences between the prevailing annotation methods^16^ and our method, so we conducted experiments to validate the feasibility of our annotation approach. Some advanced and suitable methods were employed for laser stripe image defect segmentation, and they were individually tested on our dataset. We consider that when all methods exhibit either excessively superior or excessively poor performance on the dataset, it indicates the unreliability of the dataset annotation methodology.

A total of 2,110 pairs from the multi-line laser stripe image defect segmentation dataset were used for model training, while an additional 600 pairs were used for model validation. The input resolution was set to 800 × 672, consistent with the resolution of the images in the dataset. The model training was performed with a batch size of 4 and an epoch of 60. The training optimization was conducted using the Adam method with a learning rate of 1 × 10^−2^. The cross-entropy loss function was employed for the training process.

Mean Intersection-over-Union(mIoU) is a commonly used accuracy metric for image segmentation models. The higher the value, the better the segmentation performance. IoU represents the segmentation accuracy of a specific segmentation category, while mIoU represents the average accuracy across all segmentation categories. The calculation of mIoU is as follows:

$${\rm{mIoU}}=\frac{1}{{\rm{k}}+1}\mathop{\sum }\limits_{{\rm{i}}=0}^{{\rm{k}}}\frac{{{\rm{n}}}_{{\rm{ij}}}}{{\sum }_{{\rm{j}}=0}^{{\rm{k}}}{{\rm{n}}}_{{\rm{ij}}}+{\sum }_{{\rm{j}}=0}^{{\rm{k}}}{{\rm{n}}}_{{\rm{ji}}}-{{\rm{n}}}_{{\rm{ii}}}}$$

Here, k + 1 represents the number of segmentation classes (k represents foreground classes, and 1 represents the background). n_ij_ denotes the number of pixels belonging to class i that are misclassified as class j, while n_ii_ represents the number of pixels belonging to class *i* that are correctly predicted.

The segmentation model used in the experiment is as follows: Unet^17^, BiSenetV1^18^, ICnet^19^, AGUnet^20^, DFAnet^21^, Lednet^22^, Unet3+^23^, BisenetV2^24^, Cgnet^25^.

**Fig. 9:**
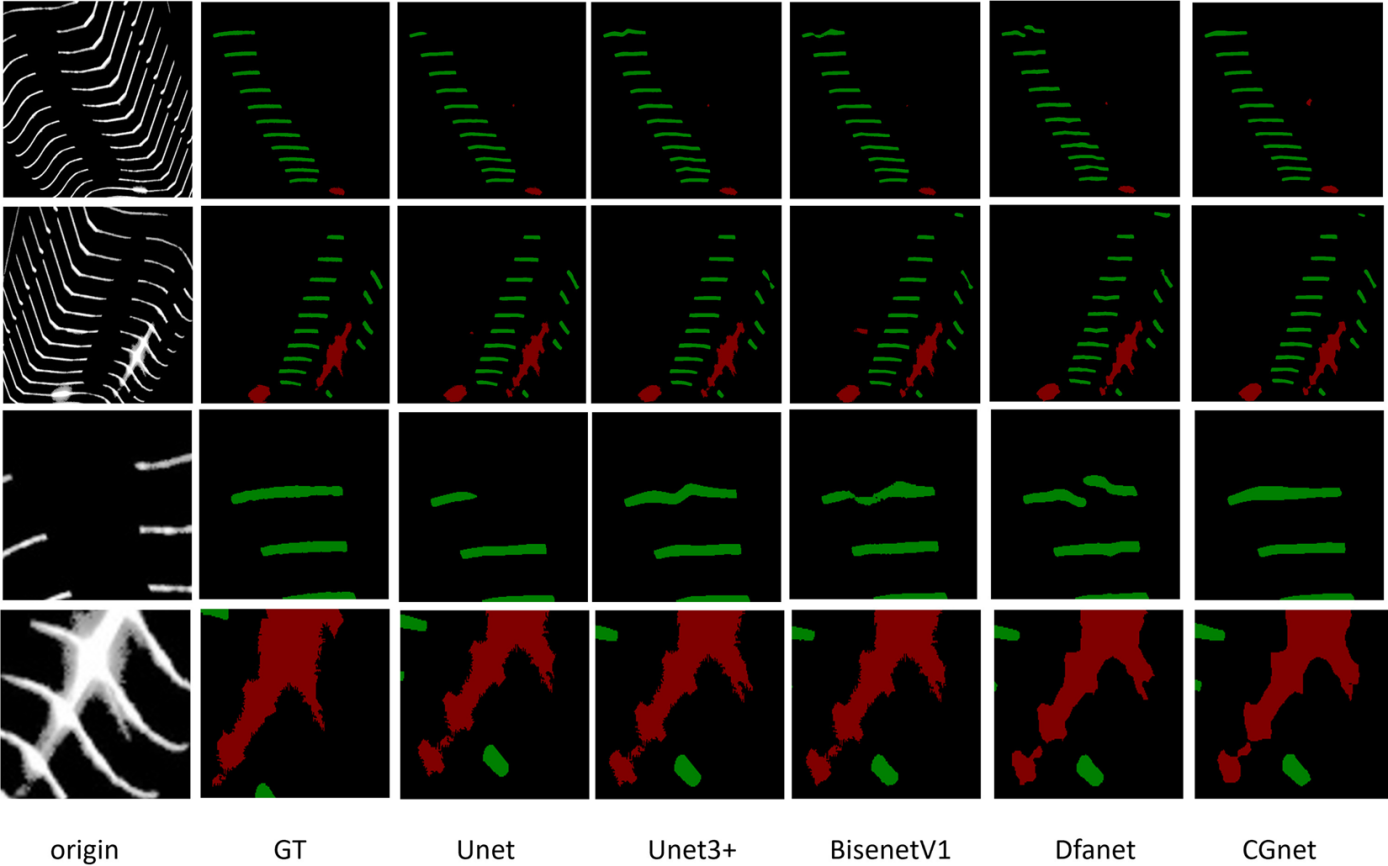
We randomly selected two images for comparison. The top two rows display the segmentation results of some models on these two images. The bottom two rows zoom in on the detailed parts, allowing readers to observe the characteristics of different models’ segmented images based on these details.

From Table 1, it can be observed that the segmentation performance for the fractured regions is generally superior to that of the light spot regions. The differences in IoU and mIoU between the models do not exceed 15%. This dataset demonstrates its reliability across the majority of classical segmentation models. Notably, several methods based on the U-Net model have shown promising results on this dataset. Researchers can start exploring methods to improve segmentation accuracy by leveraging the U-Net model. As shown in Fig. 9, we randomly selected a subset of model outputs demonstrated on the dataset. They exhibit consistency, while different models showcase distinct effects in certain details.

**Table 1:** The IoU results of 9 models on our dataset.

Model	Spot IoU/%	Fracture IoU/%	mIoU/%
U-net	90.11	94.29	92.20
BiSenet	89.19	93.10	91.15
ICnet	76.06	82.41	79.24
AGUnet	90.46	94.08	92.27
DFAnet	85.56	91.89	88.73
Lednet	84.55	92.03	88.29
Unet3+	90.04	94.30	92.17
BiSenetV2	86.13	93.37	89.75
Cgnet	84.30	92.09	88.20

### Usability validation of inpainted dataset

For the purpose of repairing laser stripe images, it is crucial to obtain an accurate ground-truth dataset. However, it is difficult to obtain such a dataset under practical industrial conditions. The primary source of spots comes from the laser undergoing diffuse reflection in the air with suspended dust particles. The main source of fractures in the stripe occurs when the laser is deflected by rapidly moving train wheels. Therefore, we captured images of wheel pairs from a low-speed unloaded train in the same conditions during dark nighttime. These images provide flawless ground-truth laser stripe images.

In reality, the shape of the wheelset differs between low-speed unloaded and high-speed loaded conditions. Therefore, the images obtained through this method can only serve as an auxiliary for repairing the fringe image, rather than being directly used for wheelset 3D measurement and reconstruction. Compared to fringe defects, the minor deformations of the wheelset can be ignored, making them suitable as reference images for repairing defective stripes. We refer to these types of images as “inpainted images”.

### Reliability of centerline extraction

In the process of extracting the centerline, we use the centerline extraction algorithm that comes with the HALCON software. Some traditional methods^26–29^ for centerline extraction can also be applied to our dataset. HALCON is based on these methods but has been improved, making it the most efficient centerline extraction software currently used in the industry. The HALCON version we use is 21.11, and we have written a code that can be used to extract the centerline.

### Error rate

In real-world applications, the maximum operating speed of heavy-haul freight trains is approximately 100 km/h. Our camera systems captured the wheelset images in this dataset while the train was traveling under a speed of 70 km/h. We ensured the image quality to control measurement errors below the standard error values specified by the national regulations. After calculations, the diameter error of the wheels was found to be less than 0.6 mm, and the errors in flange thickness and flange height were below 0.3 mm.

## Data Availability

No custom code was used during this study for the curation and validation of the dataset.
